# The Role of Bacteria, Probiotics and Diet in Irritable Bowel Syndrome

**DOI:** 10.3390/foods7020013

**Published:** 2018-01-26

**Authors:** Ashton Harper, Malwina M. Naghibi, Davinder Garcha

**Affiliations:** Protexin, Medical Affairs, Probiotics International Ltd., Lopen Head, Somerset TA13 5JH, UK; malwina.naghibi@protexin.com (M.M.N.); davinder.garcha@protexin.com (D.G.)

**Keywords:** microbiota, probiotics, irritable bowel syndrome (IBS)

## Abstract

Irritable bowel syndrome is a highly prevalent gastrointestinal disorder that threatens the quality of life of millions and poses a substantial financial burden on healthcare systems around the world. Intense research into the human microbiome has led to fascinating discoveries which directly and indirectly implicate the diversity and function of this occult organ in irritable bowel syndrome (IBS) pathophysiology. The benefit of manipulating the gastrointestinal microbiota with diet and probiotics to improve symptoms has been demonstrated in a wealth of both animal and human studies. The positive and negative mechanistic roles bacteria play in IBS will be explored and practical probiotic and dietary choices offered.

## 1. Introduction

Irritable bowel syndrome (IBS) is the most common functional gastrointestinal disorder (FGID); a group of conditions more clearly defined as “disorders of gut–brain interaction”, owed to evidence of disruption in numerous shared signalling pathways between the central nervous and gastrointestinal systems. The worldwide prevalence of IBS is 10–25% [1], which equates to between 760 million and 1.9 billion people. The UK prevalence is estimated between 10–20% [2], approximately 6.5–13 million people [3], which helps to explain why this syndrome accounts for up to 50% of visits to general practitioners for gastrointestinal (GI) complaints [2]. Understandably, the economic burden on healthcare systems from direct medical expenses and ineffective treatment are enormous, with an estimated cost of €41 billion per annum in the EU alone [4].

Numerous pathophysiological mechanisms have been explored in IBS, but the contribution of the gastrointestinal microbiota (the assemblage of microorganisms present in a defined environment) [5] and variations in its composition and function have only recently begun to be appreciated as a significant component in the pathogenesis and pathophysiology of this common syndrome. This review will explore the role of bacteria and diet in IBS pathophysiology, relevant probiotic mechanisms, and a summary of the evidence from human clinical trials.

### 1.1. IBS Diagnosis and Classification

Although the phenotype of IBS is markedly heterogeneous common symptoms include abdominal pain, altered bowel habits, bloating, and/or distension. In conjunction with this, diagnosis is widely acknowledged to rely upon the absence of any specific or unique organic pathology, such as in inflammatory bowel disease (IBD) or coeliac disease; however, developments in microbiota research are beginning to challenge this assertion. The Rome Foundation, an independent body dedicated to assisting in the diagnosis and treatment of FGIDs, published the “Rome IV criteria” for the diagnosis of IBS in May 2016. Criteria include recurrent abdominal pain (on average, at least 1 day/week in the last 3 months) associated with two or more of the following: related to defecation, associated with a change in frequency of stool, associated with a change in form (appearance) of stool. This must be fulfilled for the last 3 months with symptom onset at least 6 months before diagnosis. IBS is further categorised into diarrhoea predominant (IBS-D), constipation predominant (IBS-C), mixed type (IBS-M), and unclassified (IBS-U) based on stool form percentage using the Bristol Stool Scale (1–7—the smaller the number the firmer the stool) such that having >25% of bowel motions classified as diarrhoea would identify IBS-D, and >25% hard stools IBS-C. The usefulness of these subtypes is contentious, given that within 1 year, 75% of patients change subtypes, and ~30% switch between IBS-D and IBS-C. Furthermore, mainly for the purposes of clinical trials, the severity of IBS may be determined using the IBS severity scoring system (IBS-SSS) into mild, moderate, and severe [6].

### 1.2. The Microbiota in Health and Disease

The European Metagenomics of Human Intestinal Tract (MetaHIT) project identified that the adult GI tract is home to a staggering diversity of bacterial species (>1000) carrying a gene set more than 150 fold larger than the human genome [7]. This revelation of the vast complexity of the human microbiome has been made possible by the development of next-generation sequencing technologies [8]. Shotgun sequencing of microbial DNA extracted from a sample (metagenomics) [5] enables researchers to answer the tantalizing question “what are our bacteria capable of?”, which supersedes the ability of earlier 16S rDNA sequencing that can only identify the species present in a sample. Metagenomic studies have identified numerous factors that shape the human microbiota profile, including diet, age, geography, medications, and environment [8]. The wide individual variation in these factors is likely responsible for the heterogeneous microbiota of humans, and the resultant failure to define a uniform “healthy” configuration [9]. However, disruption in bacterial diversity and composition—commonly referred to as “dysbiosis”—is observed in numerous seemingly disparate conditions, ranging from IBS and IBD to obesity and even autism, the relative abundance and distribution of bacterial species being more similar among healthy individuals [10]. Furthermore, genome-wide association studies (GWAS) may be utilised to identify what impact genetic variation in the host has on the microbiota. Davenport et al. identified at least eight bacterial taxa associated with at least one single nucleotide polymorphism (SNP) at a genome-wide significance level [11]. In this study, the abundance of the genus *Akkermansia* was associated with SNPs within the gene PLD1—a gene previously implicated in body mass index. In support of this, a mouse study found that an increased abundance of *Akkermansia musiniphilia* was protective against developing obesity [12]. Additionally, a GWAS cohort study in a general population of Swedish twins identified that SNPs within the KDELR2 gene were associated with higher risk of IBS [13]. These correlations between bacterial taxon, genetic variation, and host phenotype are intriguing, and offer exciting new possibilities to improve human health and wellbeing through microbial manipulation.

### 1.3. Probiotics, Prebiotics, and Synbiotics

The World Health Organisation define probiotics as “*live microorganisms, which when taken in adequate amounts, confer a health benefit on the host*”. Probiotics, derived from the Latin “for life”, have been used in fermented foods for millennia. The ancient Egyptians are known to have consumed fermented milk products, Laban Rayeb and Laban Khad, as early as 9000 years ago, with fermentation growing in popularity due to its ability to preserve food and also possibly as a digestion aid [14]. Indeed, fermented foods such as bread, cheese, beer, wine, and fermented vegetables persist today. However, it has only been in more recent times that fermentation has been linked to microorganisms. Building upon Louis Pasteur’s work in the 1870s demonstrating that lactic acid fermentation was driven by microorganisms, in the early 1900s the Russian scientist Elie Metchnikoff hypothesised on the potential of altering the gut microbiota by consuming certain foods. In particular, Metchnikoff stated that consuming yoghurt would lead to *Lactobacilli* presence in the intestines, inhibiting the growth of putrefying bacteria and thus preventing “intestinal autointoxication”—poisoning with toxic substances formed within the body, as during intestinal digestion [15]. The advent of antibiotics in the early 20th century was a profound scientific breakthrough, which has saved countless lives threatened by a range of pathogenic organisms. Despite their unquestionable benefit to modern medicine, their ubiquitous and frequently indiscriminate use, combined with a deficit of new antibiotics, has led to the current global health threat of antibiotic resistance [16]. Moreover, antibiotic therapy is also associated with a number of unfortunate side effects, including *Clostridium difficile*-associated diarrhoea and candidiasis, and the possible development of a number of chronic diseases including irritable bowel syndrome, obesity, allergies, and even psychiatric disorders [17]. In recent years, there has been a growing awareness of the benefits of supplementing antibiotic therapy with probiotic therapy to prevent such illness, in a nod to Metchnikoff’s hypothesis [18].

Probiotics may exert their beneficial effects on the host through various mechanisms: (1) Pathogen suppression—competition for nutrients and mucosal space, and the production of bacteriocins (anti-bacterial proteinaceous toxins); (2) Improvement of barrier function—tight junction (spaces between adjacent epithelial cells) homeostasis; (3) Immunomodulation—pathogen-associated molecular patterns (PAMPs) are sensed by dendritic cells which influence B and T cell regulation; and (4) Neurotransmitter production—a number of lactic acid bacteria are capable of producing serotonin and gamma-aminobutyric acid (GABA) which may influence the communication between the gut and brain (gut–brain axis) [19].

#### 1.3.1. Prebiotics and Synbiotics

The latest consensus definition of prebiotics by the International Scientific Association for Probiotics and Prebiotics defines them as “*a substrate that is selectively utilised by host microorganisms conferring a health benefit*” [20]. Prebiotics such as inulin and inositol are found in foods including leeks, asparagus, Jerusalem artichokes, garlic, and onions [21]. Some of the most common prebiotics are described below.

▪ Inulin: Belonging to the fructan family of dietary fibres, inulin is non-digestible. It is therefore able to pass through the small intestine intact and reach the colon. Here, inulin is fermented—particularly by *Bifidobacterium* species and other lactic acid-producing bacteria—boosting the numbers of these health beneficial bacteria [21]. Fermentation products of inulin offer colon cancer-preventing properties, and short chain fatty acids (SCFAs) are also produced during fermentation.
▪ β-glucan: This exopolysaccharide is found naturally in cereal grains, bacteria, and fungi [22]. It has been shown to have prebiotic properties on the growth of *Bifidobacterium* and *Lactobacillus* species [23]. Fermentation of β-glucan by *Bifidobacterium infantis*, in particular, was shown to increase the production of SCFAs [24].
▪ Fructooligosaccharides (FOSs): Present in wheat, honey, onion, garlic, and banana, FOSs are short-chain carbohydrates which resist digestion in the small intestine. In the colon, they promote *Bifidobacterium* and are converted to SCFAs, and also contribute to faecal matter, improving bowel movement. Conversely, they inhibit the pathogen *Clostridium perfringens* in the colon [25].

Synbiotics are products containing both prebiotics and probiotics, whereby they act synergistically. In a synbiotic, the prebiotic component is designed to selectively stimulate either growth or metabolism by the probiotic bacteria, and may therefore be considered for use where there may be survival challenges for the probiotic alone [26]. In addition, they may also stimulate certain commensal bacteria in the GI tract [27]. In humans, synbiotics have a range of effects, including increasing numbers of health beneficial bacteria, improved immunomodulation, and reduced incidence of nosocomial infection in patients post-surgery [27].

#### 1.3.2. Probiotic Regulations and Product Variety

The variety of health beneficial bacteria products on the market today is dizzyingly vast, and not all of these bacteria are classified as “probiotics”—a situation that presents a significant challenge of appropriate choice for both consumers and healthcare practitioners. The regulations governing the introduction of probiotics to the market vary by geographical region. The European Food Safety Authority (EFSA) is requested to assess the safety of a broad range of biological agents, and produces an annually updated list of microbes intentionally added to foods (QPS, Qualified Presumption of Safety of Micro-organisms in Food and Feed, list) [28]. This document lists organisms as far as the species level that are considered safe to enter the food chain. In the USA the equivalent of the QPS is GRAS (Generally Recognized as Safe)—a designation regulated by the American Food and Drug Administration. Novel probiotic strains should have complete genomic sequencing, antibiotic resistance, and toxicology assessments performed. Once a microbial species has satisfied these safety assessments, there are further important factors that differentiate probiotic products:

▪ “Strain specificity”—not all strains of the species *Lactobacillus rhamnosus* are genetically exactly the same, and therefore may not share the same functionality. Douillard et al. showed that strains of the same species obtained from different sources have a significant variability in their resistance to bile acid; dairy sourced strains were more susceptible than human isolates [29].
▪ Diversity of included strains—single-species (just one type of bacterium), multi-strain (this often refers to not only multiple strains, but multiple species and even multiple genus formulations).
▪ Preparation—food ingredient, liquid medium, compressed tablet, or freeze-dried (lyophilised) powder (sachet or capsule).
▪ Dosage—there is a wide range of product colony forming unit (CFU) dosages; e.g., 100 million to 450 billion CFU per dose.
▪ Probiotic or synbiotic—does the product only contain probiotic strains, or are they in combination with other ingredients such as prebiotics—referred to as synbiotics.

### 1.4. Sex, Genes, and Bacteria in IBS

Relatives with a family history of IBS have a 2–3-fold increased risk for IBS, and estimations based on twin studies suggest that genetic heritability of IBS ranges from 22–57%, with higher prevalence in monozygotic compared to dizygotic twins [30]. In Western populations, women are more frequently diagnosed with IBS than men in a ratio of ~2:1. Female predominance in IBS appears around the time of puberty, rises during early adulthood, and then declines with age reaching a similar prevalence in both sexes by the seventh decade of life—a pattern that implicates the involvement of reproductive hormones [31]. Interestingly research has shown that sex hormones directly affect the composition and metabolism of the microbiota. The gut microbiota has been shown to change dramatically between the first and third trimesters [32], and male and female rats produce a different profile of short-chain fatty acids when fed the same diet [33]. Furthermore, in a murine model oestrogen receptor-β (ERβ^+/+^) animals had a significantly greater proportion of *Bacteroidetes* (ERβ^+/+^ = 28.7%; ERβ^−/−^ = 20.4%; *p* = 0.004) and a significantly lower proportion of y-*Proteobacteria* (ERβ^+/+^ = 13.8%; ERβ^−/−^ = 21.0%; *p* = 0.001) than ERβ^−/−^ mice [34]. ERβ status therefore plays a role in the selection of intestinal microbiota, which may have numerous consequences for gut and extra-intestinal homeostatic mechanisms.

Specific genetic disturbances related to 5-hydroxytryptamine (5-HT, serotonin) have been identified in IBS [30]. The function of the serotonin transporter (SERT)—from gene SLC6A4—is to remove 5-HT from the synaptic cleft back into the presynaptic neuron, thus terminating its action. Both IBS-D and IBS-C genotypes have been associated with short allele polymorphisms of the promoter length polymorphism 5-HT transporter-linked polymorphic region (5-HTTLPR), which can affect SLC6A4 transporter expression [35]. SERT variants such as SERT Ala56—which has been identified in children with autism spectrum disorders (ASDs)—confers an increased 5-HT transport relative to wildtype SERT [36]. Introduction of this mutation in mice resulted in typical ASD behaviours. These mice were also constipated and had bacterial intestinal overgrowth, again commonly seen in ASD [37]. Thus, alterations in serotonin transport may disrupt gastrointestinal homeostasis, leading to dysbiosis and a resultant IBS phenotype; however, our microbiome may also influence 5-HT homeostasis. The vast majority of serotonin is located in the gut, where it is produced from the precursor molecule tryptophan within enterochromaffin cells [38]. The dominant physiological pathway for tryptophan is however down the kynurenine pathway, involving the enzymes tryptophan 2,3-dioxygenase (TDO) and indoleamine 2,3-dioxygenase (IDO). Once kyneurinine is produced from tryptophan, it is further metabolised into either the neuroprotective kynurenic acid (KYNA) or the neurotoxic quinolinic acid (QUIN). The TDO enzyme is activated by glucocorticoids (stress) and the IDO enzyme by pro-inflammatory cytokines (inflammation). Activation of these enzymes may reduce the availability of tryptophan for serotonin synthesis whilst also altering the balance between neurotoxic and neuroprotective metabolites. The study by Valladares et al. showed that a *Lactobacillus* strain fed to rats significantly reduced kynurenine with a concomitant rise in 5-HT levels [39]. Hydrogen peroxide (H_2_O_2_) produced by the bacteria arrested IDO activity in vitro, and *Lactobacilli*-fed animals were found to have a nearly four-fold increase in luminal H_2_O_2_ content, thus providing a potential mechanism of action. Interestingly male IBS patients were found to have increased IDO activity in conjunction with elevated levels of neopetrin [40] (a product of macrophages that indicates a pro-inflammatory immune status) compared to controls. A pro-inflammatory immune status in IBS patients may thus be affecting tryptophan metabolism by activating the immunoresponsive IDO enzyme.

### 1.5. Environmental Risk Factors and the Microbiota in IBS

IBS accounts for 60% of pain-related FGIDs in children [41]. Given that IBS is relatively common in childhood, it has been hypothesised that early life events may play a part in its development. In a multivariate model, Koloski et al. (2015) identified that early hygiene factors (sharing a bedroom and exposure to herbivorous pets) up to age 5 years (odds ratio (OR) = 8.93, *p* = 0.03) and female gender (OR = 3.34, *p* < 0.001) were both significant independent predictors of IBS in later life. Interestingly, in the same study, a shorter duration of breastfeeding was significantly associated with IBS as an adult (5.6 months vs. 8.1 months, *p* = 0.009), and although not significant, there was also a trend for higher numbers of cesarean deliveries in IBS [42]. These findings are relevant to the role of the microbiota in IBS, given that research has shown a significant difference between the bacterial colonisation pattern between babies that are breastfed and formula-fed, and also between those that have cesarean versus vaginal birth [43]. Furthermore, *Bifidobacterium* and *Lactobacillus* species are enriched in breastfed infants compared to those exclusively fed formula milk; notably, these species are found to be reduced in adult IBS patients, thus highlighting their potential significance to the syndrome.

### 1.6. Dysbiosis in IBS

IBS subjects have a demonstrable alteration in their intestinal microbiota compared to healthy controls [44]. Studies have identified both a general decrease in diversity [45], and more specifically a decrease in *Bifidobacterium* and *Lactobacillus* species abundance [1], and an increase in *Gammaproteobacterium* species [46]—a class containing numerous prominent pathogens. In a recent study, significant decreases in *Roseburia* (a predominant butyrate-producing genus) and *Bifidobacterium* species were measured in IBS-C patients, whilst sulphate-reducing bacteria (SRBs) were found many-fold higher compared to controls [47]. SRBs produce toxic sulphide compounds, which have been shown to play a role in visceral nociception and colonic motility, leading the authors of this study to conclude that the dysbiosis observed may be responsible for symptomatology. Furthermore, an inverse correlation between *Bifidobacteria* and abdominal pain was identified by Jalanka-Tuovinen et al. [48], such that subjects who experienced pain had over five-fold less *Bifidobacteria* compared to those without pain, thus highlighting the possible importance of this genus in visceral hypersensitivity.

## 2. Evidence of Bacterial Disturbance Causing IBS

### 2.1. Post-Infectious IBS

Post-infectious IBS (PI-IBS), which bears close resemblance to IBS-D, is a surprisingly common result of acute gastroenteritis (primarily of bacterial aetiology) with a reported incidence of between 5–32% [49]. The suggested pathophysiologic mechanisms here include increased intestinal permeability, altered motility, and persistent intestinal inflammation [49]. Research published in 2015 showed that host antibodies to a toxin—cyto-lethal distending toxin B (CdtB)—produced by *Campylobacter jejuni* cross-react with vinculin (a host epithelial cell adhesion protein), the presence of which predicted progression to an IBS-like phenotype [50]. The animals in this experiment had changes in stool form, increased rectal intra-epithelial lymphocytes (also seen in humans with IBS), and levels of anti-CdtB correlated with the levels of small intestinal bacterial overgrowth (SIBO). Intriguingly, anti-CdtB titers were significantly higher in D-IBS subjects compared to non-IBS subjects (*p* < 0.001), therefore potentiating its usefulness as a future biomarker in the workup of chronic diarrhoea.

### 2.2. Small Intestinal Bacterial Overgrowth and IBS

Small intestinal bacterial overgrowth (SIBO) is defined by an excessive amount of bacteria in the small intestine, and may be a cause of IBS. SIBO arises when the homeostatic mechanisms that regulate enteric bacterial populations are compromised. Small intestinal dysmotility and reduced gastric acid secretion are the most common predisposing processes. Studies in IBS patients have revealed delayed transit, intestinal dysmotility, and abnormalities in the migrating motor complex—all of which could account for a predisposition to SIBO [51]. However, determining SIBO prevalence from IBS studies is hindered by conflicting results, and the validity of current routine diagnostic tests (culture and glucose and lactulose-hydrogen breath tests) have been criticised for their lack of both sensitivity and specificity [52]. The reduction in microbial richness shown with rifaximin therapy may be responsible for its modest clinical benefit in diarrhoea-predominant IBS patients, but SIBO was not tested in these patients and the underlying mechanism of action remains to be clearly defined. Determining the role small bowel bacteria play in IBS pathophysiology will require large-scale studies that apply culture-independent molecular testing that are capable of a more detailed exploration of host–gut microbial cross-talk.

### 2.3. Antibiotics and IBS (Iatrogenic IBS)

Antibiotics significantly alter gut microbial ecology (namely a collapse in diversity), and disrupt interactions with host metabolism [53]. A consequence of this is the frequent disturbance in bowel habit, particularly with broad-spectrum antibiotics (5–62%) [18], and numerous animal and human studies provide evidence of the role antibiotics play in IBS pathogenesis. Early life exposure to antibiotic alters microbial composition and leads to enduring effects on visceral pain in rodents [54]. Evidence that this also occurs in adulthood was shown in a prospective study in which consecutive patients prescribed antibiotics for non-GI complaints were more than three times as likely to report chronic bowel symptoms as controls [55]. Moreover, a large GP survey of over 400 patients, aged 18–80 years, found that antibiotic usage was strongly associated with IBS symptoms (OR 3.70). Interestingly, probiotics—in rodent studies at least—can ameliorate visceral pain induced by antibiotic administration [56].

## 3. IBS Symptoms and Bacteria

IBS has traditionally been viewed as a complex involving one or more of: visceral hypersensitivity, gastrointestinal dysmotility, and psychopathology.

### 3.1. Bacteria and Visceral Hypersensitivity

Animal studies have shown that development in germ-free conditions (an absence of bacterial colonisation in the gut) leads to visceral hypersensitivity [57], which may be normalised by the later introduction of gut microbes, thus providing evidence that bacterial colonisation is required for the normal development of nociceptive physiology. A recent study also showed that colonic hypersensitivity can be transferred to rats through faecal transplants from IBS patients [58]. Visceral hypersensitivity is a salient pathophysiological feature of IBS which may involve disruption in any or all of immune, neural, endocrine, and metabolic processes [1]. Immune pathways may be triggered by increased intestinal permeability—a prevalent feature of IBS-D that corresponds to the severity of symptoms [59]. Intestinal permeability is affected by probiotics through their positive influence on a range of tight junction proteins, which hold intestinal epithelial cells together [44]. Interestingly a multi-strain probiotic was shown to significantly reduce intestinal permeability in patients with IBS-D [60]. In a further trial, IBS patients were found to have an abnormal pro-inflammatory IL-10/IL-12 ratio, which was normalised—in conjunction with symptom alleviation—by the consumption of the probiotic *Bifidobacterium infantis* 35624 [61]. Neural mechanisms involved in visceral pain perception also seem to be influenced by certain bacterial species, such as the induction of μ-opioid and cannabinoid receptors by *Lactobacillus acidophilus* [62].

### 3.2. Motility and Bacteria in IBS

Gastrointestinal dysmotility—a prominent feature of IBS—manifests in delayed transit in patients prone to constipation and in accelerated transit in patients prone to diarrhoea. The relationship between bacteria and intestinal motility is seen in intriguing research into Toll-like receptors (TLRs), which are known to control intestinal epithelial homeostasis. TLRs are transmembrane receptors that are involved in innate immunity, pain modulation, and gastrointestinal motility [63]. A variety of receptors exist in mammals that are capable of detecting different pathogen-associated molecular patterns (PAMPs), such as lipopolysaccharide (LPS) by TLR4 and flagellin by TLR5 [64]. LPS are components of the outer membrane of gram-negative bacteria, which are abundantly found in the colon. Animal research in this area has identified that a low level of LPS is essential to maintain neuronal survival, however at higher doses LPS results in neuronal toxicity [63]. In TLR4 mutant and TLR4 knock-out mice, a significant delay in gastrointestinal motility is observed [63]. It is perhaps not surprising that patients with IBS-D have been found to have an increased expression of TLR4 from colonic biopsies [64]. Moreover, this receptor is increased roughly 15-fold in inflammatory bowel disease patients. Indeed, LPS and flagellin have both been shown to upregulate TLR4 and TLR5, respectively; thus, the increased expression of these receptors may be in reaction to increased levels of these bacterial components. In a human study, Zhou et al. showed that a high-FODMAP diet (fermentable oligo-, di-, and monosaccharides, and polyols) increased faecal LPS, potentially as a result of dysbiosis [65], which induced mucosal inflammation, increased permeability, and contributed to visceral hypersensitivity [12]. A greater abundance of gram-negative bacteria in IBS patients could possibly account for this, or potentially a greater abundance in the wrong place, such as in SIBO [66], which is characterised by a colonic bacterial profile being identified more proximally within the gastrointestinal tract. To add extra complexity to this situation, there is evidence that not all LPS are created equally. The tetra- or pentacylated lipid A of the *Bacteroidetes* (abundant colonic commensal phylum) LPS seems not to be agonistic to TLR4, whereas the hexacylated lipid A of *Enterobacteriaceae* (a family within the *Proteobacteria* phylum that are 4–6-fold less abundant than *Bacteroidetes* in humans) is agonistic [67]. Of note, the enteric pathogens implicated in post-infectious IBS (PI-IBS)—which most commonly results in a diarrhoea predominant phenotype—include *Campylobacter, Salmonella, Escherichia coli*, and *Shigella* species—all of which are members of the *Proteobacteria* phylum, and all producing hexacylated lipid A. A hypothetical benefit of probiotics or dietary changes may therefore be in reducing *Proteobacteria*, the concentration of agonistic LPS, and thus ameliorating motility disturbances in IBS. In support of this, *Lactobacillus paracasei* has been shown to attenuate gut muscle hypercontractility in an animal model of post-infectious IBS [68].

### 3.3. Psychopathology and IBS

Increasing research is revealing the interaction between psychiatric disorders including generalised anxiety disorder, major depressive disorder, and schizophrenia and IBS [69]. Both animal and human studies have demonstrated a link between altered gut microbiota and depression [10]. Worryingly, a UK study identified that IBS has the potential to cause fatal outcomes from suicide, with depression not accounting for all the variance in suicidal ideation [70]. Probiotics capable of conferring mental health benefits through interactions with commensal gut bacteria have been coined “psychobiotics” [71]. The psychophysiological effects of psychobiotics include psychological effects on emotional and cognitive processes, systemic effects on the hypothalamic–pituitary–adrenal axis, and neural effects via neurotransmitters and neurotrophic proteins [71]. In a human study using functional magnetic resonance imaging (fMRI), 4 weeks of a multi-strain probiotic altered emotional processing compared to placebo [72]. Germ-free (GF) mice have an exaggerated stress response with heightened levels of stress hormones—a finding that was reversed by treatment with *Bifidobacterium infantis* [73]. Furthermore, low plasma serotonin (5-HT) in GF animals can be normalised and augmented with probiotic bacteria [71]. Taken together, there is a suggestion that our gut microbiota may be influencing central nervous system homeostasis, thus offering an explanation for the common association of dysbiosis and psychopathology in IBS whilst also offering a therapeutic target for probiotic treatment.

## 4. Clinical Evidence of Probiotics in the Management of IBS

The complex interplay between genetic, microbial, and environmental factors is likely responsible for the phenotypic heterogeneity in IBS, and the successful treatment of different subsets may therefore require specific microbial manipulations. Host specificity has been suggested to be a desirable property for probiotic bacteria, and is therefore recommended as one of the selection criteria [74]. A comparative genomic and functional analysis of 100 *Lactobacillus rhamnosus* strains identified that the production of functional mucus binding pili (SpaCBA) was significantly more prevalent in human isolates (40.2% or 31/77) than in dairy isolates (13% or 3/23) [29]. The authors suggested that the human-mucus binding pili may provide a colonization advantage in the intestinal tract, which may be lost in strains evolving in other ecological niches, such as dairy products. Clinical trials that utilise strains selected from non-human ecological niches may therefore be at greater risk of failing to treat IBS symptoms, as they are not adapted to the human gastrointestinal environment. This may be the reason for the variable results observed in human clinical studies in IBS with probiotics to date [75]. A 2015 meta-analysis of 24 human clinical trials concluded that probiotics, overall, were more beneficial than placebo in reducing pain and symptom severity scores [76]. A 2016 systematic review produced by the British Dietetic Association (BDA) analysed the findings of 35 human randomised controlled trials, of which 83% improved at least one outcome [77]. The authors concluded that, given the heterogeneous results of the included studies, specific recommendations for IBS management were not possible, which we are in agreement with. However, if we analyse the results in terms of probiotic features such as single versus multi-strain trials and concentration (CFU/day), it is possible to utilise the data to make some informed choices.

### 4.1. Multi-Strain versus Single-Strain Probiotics in IBS

The analysis included 16 single-strain and 19 multi-strain products. Of the studies that found a statistically significant improvement in “global symptoms” (14 out of 29 studies) and a clinically meaningful improvement in “abdominal pain” (only 8 of all 35 trials), the majority were multi-strain products (~2:1). Furthermore, only multi-strain trials found a clinically meaningful improvement in quality of life in IBS patients [78,79]. The mechanisms underlying this difference in efficacy remain to be defined, and are likely complex. However, in an in vitro intestinal epithelial cell inflammation model, MacPherson et al. demonstrated that, in comparison to single-strains, a multi-strain challenge resulted in a greater reduction of inflammation-modulated genes—findings that indicated a synergistic effect of bacterial combinations in resolving inflammation and maintaining cellular homeostasis, which the authors concluded reinforces the rationale for using multi-strain probiotic formulations [80]. We could hypothesise therefore that the more frequent success of multi-strain probiotics in IBS is owed to a synergistic effect on disease-modulated genes.

### 4.2. Probiotic Concentration in IBS

Of the 35 trials analysed in the BDA review, only 14 showed a clinically meaningful result in at least one outcome measure including global symptoms, pain, bloating, or quality of life (QoL). Nine of the fifteen studies used a daily dose of 10 billion CFU or less, while only five used more than 10 billion CFU (Table 1). Moreover, four of the five largest successful studies utilised a probiotic with a concentration <10 billion CFU (Table 1). A 2017 review of probiotic dose–response studies, for a range of indications, only found a compelling relationship in antibiotic-associated diarrhoea prevention [81]. Interestingly a 2016 meta-analysis of probiotics in IBS concluded that low-dose probiotics were more effective in reducing overall symptom response and QoL [82].

Based on the available evidence from the BDA review, it seems that a multi-strain probiotic with a concentration of 10 billion CFU/day or less may have the best chance of providing a clinically meaningful outcome for IBS sufferers. However, there is clearly a need for further large well conducted randomised controlled trials to provide more reliable results to inform probiotic choice for treatment of this syndrome.

## 5. Diet, Bacteria, and IBS

Less than 40% of IBS patients are satisfied with their current treatment [94]; therefore, alternative self-management techniques are explored such as relaxation (e.g., yoga) or dietary changes (e.g., low FODMAPs). Diet was seen as a main culprit in IBS from the onset, as it predominantly presents with gastrointestinal symptoms. Over the last 30 years, researchers have tried to understand how different foods exert deleterious effects on IBS patients.

### 5.1. New Diets, Old Genes

Diet, lifestyle, and environment have rapidly changed in the last two centuries, while our genetic makeup has not altered appreciably over the last 200,000 years. This situation has often been blamed for “diseases of civilization”, one of which is IBS. Dairy, cereals, refined sugars, refined vegetable oils, and alcohol make up over 70% of total daily energy consumed by an average US citizen, which is drastically different to the diet of our pre-agricultural ancestors (approximately 10,000 years ago), where these types of food would have contributed in marginal amounts, and over 50% of food would be animal-based [95]. It is not only the difference in food quantity, but also in its quality. Until about 150 years ago, cereals would be made using the whole grain (including germ, bran, and endosperm), while now it is highly refined. For millennia, fruits and honey—main sources of simple sugars—were consumed in high amounts, but restricted by seasonality, and did not influence overall energy intake. Change in food production in the last 100–200 years has set an unprecedented situation where sugar, salt, and meat fat are consumed all year round in unlimited amounts. This change in diet and clash with genetic setup has a number of health ramifications (i.e., change in glycaemic load, micro- and macronutrient density, sodium-to-potassium ratio, or fibre intake) [96]. Therefore, one path of understanding diseases demonstrated by gastrointestinal symptoms would be to understand the consequences of the imbalance between our genetic makeup and lifestyle. However, other than implementing a restrictive diet like our pre-agricultural ancestors (e.g., paleo diet), we are beyond reversing changes that took place over the last centuries.

### 5.2. IBS Symptomatology and Diet

Exclusion diets are predominant dietary interventions for IBS. When introduced by a dietitian, they can be followed up by re-challenge to evaluate change in tolerance, while others require longer-term reduction of the targeted food component. Research shows that 70% of IBS patients report symptoms related to 19 categories of foods, and the majority of those patients reduced intake of up to 14 food categories, mostly milk and cheese, onion, cabbage, red meat, and alcohol—especially beer [97]. Exclusion of dairy products in almost 35% people carries a risk of nutrient deficiency, especially calcium. Perceived intolerance to dairy products was however much higher than confirmed lactose malabsorption, or presence of milk antibodies (evident in 6 out of 35 individuals) [97]. This low agreement between physiological response to food and perceived intolerance and symptoms stimulation suggests that the link between food and IBS symptoms is not direct. Similarly, the response to alcohol consumption differed depending not only on the consumed amount, but also IBS type, with female IBS-D patients suffering the most severe symptoms a day after [98]. Currently available evidence of the role of coffee, alcohol, or dairy in the IBS symptomatology is based on cross-sectional observational studies, and to date no randomised control trials (RCTs) have been published on the effectiveness of such dietary restrictions.

Amongst various dietary interventions for IBS, the most popular include a modified fibre diet or exclusion of FODMAPs, gluten, carbohydrates, fructose, or restriction of caffeine or capsaicin (component of chili peppers). In 2017, Tuck and Vanner reviewed the available dietary therapies to treat symptoms in patients with IBS [99], and Table 2 presents a summary of their findings.

One of the first and most studied dietary modifications focused on fibre, and several systematic reviews of various RCTs have been published, without coherent conclusion since soluble fibre and insoluble fibre appeared to have different effects [100]. In addition, it must be considered that fibre may act as prebiotics for not only beneficial commensal and transient species, but also opportunistic and pathogenic strains; therefore, an increased prebiotic intake in individuals with dysbiosis may have deleterious effects [101].

From the initial concept of fibre restriction, a new approach was identified where a diet low in fermentable carbohydrates (low-FODMAP diet) was developed. A number of RCTs have explored the efficacy of a low-FODMAP diet [102,103,104], suggesting that up to 70–80% IBS patients have seen reduction in symptoms severity, including reduced abdominal pain and flatulence—two core symptoms of all IBS subtypes [94]. The availability of FODMAPS for fermentation could be due to (a) absence or reduced concentration of enzymes that would hydrolyse the carbohydrate (e.g., lack of lactase leads to lactose availability in the colon); (b) through an incomplete absorption in the small intestine. The effect of individual fermentable carbohydrates appears to be additive and dose-dependent, and therefore a low-FODMAP diet was based on a stepwise approach with initial 4–8 weeks elimination of most FODMAP products, symptoms evaluation and graded re-introduction of individual products to build tolerance, and adaptation over time [105]. Reviews of potential mechanisms by which a low-FODMAP diet could impact IBS symptoms were published previously [102,106], and in summary this could be due to normalisation of colonic serotonin cell density or normalisation of stool lipopolysaccharide levels, which may impact visceral hypersensitivity. While there is no causal evidence and this could be merely an epiphenomena, both mechanisms are biologically plausible. The Western diet has typically up to 40 g of unabsorbed carbohydrates per day [107] (less than 18 g were shown to be FODMAP [108], the rest are polysaccharides like cellulose, pectin, psyllium), which have both favourable effects (increase stool bulk, enhance calcium absorption, provide substrate for bacteria) and negative effects (increases small intestine water volume, changes intestinal motility and bacterial gas production). Based on the assumption that higher level of unabsorbed carbohydrates results in a higher level of fermentation (greater gas production and water retention in the intestine), the low-FODMAP diet was aiming for a reduction of luminal distention [109]. In 2013, Zhu et al. demonstrated that prevalence of distention after lactose ingestion was similar in IBS and healthy [110], and Major and colleagues suggested that it was hypersensitivity to distension rather than excessive gas production that was at the core of carbohydrate-related symptoms [111]. In the latter study, challenge with fructose and inulin showed, respectively, increased small-bowel water content and increased colonic volume and gas production to a similar extent in healthy and IBS patients. Despite similar MRI parameters, IBS patients reported greater change to symptoms and peak of symptoms was aligned with peak colonic gas [111]. Therefore, the evidence suggest that a low-FODMAP diet has a beneficial effect for a large group of IBS patients, but it could be via different mechanisms than originally assumed. Several publications have recently reviewed the efficacy of the low-FODMAP diet [112], with different conclusions. Alongside potential IBS symptoms reduction, various concerns were raised, such as: diet complexity and limited evidence to guide best practice; need for assistance of dietary expert; risk of calcium deficiency; unknown long-term effects; and most recently, unclear impact on gut microbiome [106,113,114,115,116].

### 5.3. Diet as a Moderator of IBS Pathophysiology

The pathophysiology of IBS from a dietary perspective is complex and unclear, since IBS is an umbrella term for patients with various presentation and history. Therefore, no single process or component can be determined as a culprit. There is no evidence to suggest that those who developed IBS in the past had a significantly different diet from healthy individuals. However, diet and its macro- and micronutrient composition has direct (feeds bacteria) and indirect effects on the gut microbiome (alterations to pH and transit time). It has been shown that Mediterranean diet (high in fibre and antioxidants, but low in red meat) was related to higher abundance of all bacteria, including *Bifidobacteria* and *Lactobacilli*. In contrast, Western diet (high in animal fat and protein) and gluten-free diet was related with reduced total bacteria and *Bifidobacteria*, along with increased *Enterobacteria* [117]. Given the intricate relationship between IBS symptoms and diet, various dietary modifications were suggested for IBS management. It was expected that alterations to the composition of a diet—especially components directly impacting bacterial community, such as fermentable carbohydrates—would have an effect on microbiome composition, function, and by proxy, IBS symptoms.

Most dietary components can be utilised by all bacteria, since human milk oligosaccharides present in breast milk are one of few prebiotics that have been shown to be a selective growth substrate for *Bifidobacterium* species [118]. Therefore, diet high in FODMAPs—natural non-selective prebiotics—provides food not only for beneficial, but also opportunistic and pathogenic bacteria. Based on the supposition that IBS is related to dysbiosis [1], reduced supply of prebiotics was expected to alter the gut microbiota abundance. Reduction in methanogenic bacteria [119] could have a direct impact on colonic gas volume, since methanogenic bacteria utilise hydrogen and reduce gas volume by up to 75% [120].

Studies have shown that a low-FODMAP diet alters the gut microbiota in potentially negative ways, such as reducing the absolute abundance of *Bifidobacterium* spp. and increasing the richness of *Firmicutes*; specifically *Clostridiales* [106]. Therefore, the evidence suggests that short-term use of low-FODMAP diet can reduce symptoms and improve tolerance to some foods, but the long-term impact on the microbiome may have unforeseen deleterious consequences. To counteract this effect of a low-FODMAP diet, the use of probiotic supplements may serve as an appropriate adjunct; however, only one RCT has been published to-date [121]. The study incorporated a unique approach with a sham diet (similar complexity and number of changes without altering total intake of FODMAP) to mimic the restrictive nature of low-FODMAP diet (major advantage over studies comparing low-FODMAP diet with lack of dietary changes). However, a factorial design with 104 individuals categorised into four groups (sham diet + placebo, sham + probiotic, low-FODMAP + placebo, low-FODMAP + probiotic) restricts the conclusions. This study suggests that probiotics in combination with a low-FODMAP diet could prevent unfavourable changes to the microbiota; however, more evidence is required to firmly establish whether this approach is any more or less beneficial than either intervention alone.

There is an overlap between non-celiac gluten sensitivity and IBS-type symptoms, since wheat is commonly reported as one of the foods triggering IBS symptoms. There is a risk of misdiagnosis of celiac disease as IBS, and potentially non-celiac gluten-sensitive patients are a group of self-diagnosed IBS patients who self-treat by adhering to a gluten-free diet. Self-diagnosis of gluten sensitivity and adherence to gluten-free diets is common (3–5% adults), and in some groups (e.g., Australian athletes) may be as high as 40% [122]. Similar to the low-FODMAP diet, diets excluding gluten have a direct impact on the gut microbiome. Studies suggest that one month of a gluten-free diet significantly reduced *Lactobacilli* and *Bifidobacteria*, and in parallel increased the abundance of *E. coli* and total *Enterobacteriaceae* [123]. In addition, gluten appears to have a direct effect on the gut barrier. Undigested peptides remaining after incomplete degradation of gluten and some other wheat proteins can pass through a more permeable epithelial barrier, reach the submucosa, and activate the resident innate immune cells. Studies with transgenic mice sensitised by gluten demonstrated that human leucocyte antigen DQ8 (HLA-DQ8) show an altered barrier function and enhanced muscle contractility—mechanisms previously observed in IBS patients [124]. Recently, it was shown that up to 50% of IBS patients have the genetic factors (HLA-DQ2/DQ8) predisposing to gluten sensitivity [125], which was in line with the findings of Catassi et al. in a review of the effectiveness of gluten-free diet in IBS, which identified an overall positive response [122]. However, a recent RCT involving a gluten challenge showed that 49% of non-celiac gluten-sensitive individuals paradoxically developed symptoms after a gluten-free diet [126]. Skodje et al. suggested the dietary culprit may be fructan, rather than gluten, that induces symptoms in non-celiac gluten-sensitive individuals [127].

Dietary management of IBS currently focuses on common triggers and FODMAPs, while evidence suggests that various food additives could be of relevance. Commonly used artificial sweeteners (sorbitol and mannitol) not only increase FODMAP load, they also show discordant absorption compared to healthy individuals [128]. Saccharin, sucralose, or aspartame were shown to change the composition and function of the gut microbiota and drive glucose intolerance in both animal and human models [129]. A study showed that dietary emulsifiers (polysorbate and carboxymethylocellulose) used in products like ice cream can alter the microbiota, leading to increased active flagellin levels with pro-inflammatory consequences [130]. The impact of such changes have not been studied from the IBS perspective to-date, but considering the importance of the gut microbiome in IBS pathophysiology, it is important to consider any dietary component that directly or indirectly alters its function.

## 6. Discussion

A wealth of microbiome research suggests that numerous homeostatic mechanisms are influenced by the bacteria we host within. The communication between our microbiota and body systems seems to be bidirectional, and the malleable nature of the former presents an exciting target for the treatment of a range of human diseases. There is clear direct evidence of an alteration in the overall diversity and specific abundance of bacteria in IBS, although this difference is heterogeneous between IBS sufferers. We have seen the impact that sex, genetics, early environmental factors (delivery method, animal exposure, and mode of infant feeding), antibiotics, and diet can have on the microbiota and its potential association with IBS. The mechanisms that connect this observed dysbiosis and symptoms are not yet fully elucidated, however it is interesting to note the impact that pathogenic bacteria such as *Proteobacteria* may have in the origins and continued symptomatology of this condition. Probiotic and dietary interventions offer the potential to alter the community and/or metabolic output of the microbiota, and the clinical efficacy of these options is evidenced in a mounting body of research.

## 7. Conclusions

The role that bacteria play in the pathogenesis of IBS may be substantial; however, this relationship is extraordinarily complex and certainly involves numerous mechanisms which remain to be fully elucidated. Future research in this field requires statistically robust randomised controlled trials, with the ability to more definitively show both the clinical effectiveness and mechanistic basis of probiotic preparations and dietary interventions in the management of IBS. Based on current evidence, it seems that multi-strain probiotics, at a concentration of 10 billion CFU/day or less, offer the best chance of improving abdominal pain, global symptoms, and crucially, quality of life in IBS sufferers. Low-FODMAP diets also seem to be an effective option for many IBS sufferers, and whether a combination of probiotics and a low-FODMAP diet is the optimal management requires further research.

## Figures and Tables

**Table 1:** This is a table presenting those studies analysed in the 2016 British Dietetic Association (BDA) review that had a clinically meaningful outcome.

Study	Sample Size	Strains	Concentration	Duration
Ducrotte 2012 [83]	214 *	*L. plantarum* 299vd	(1 × 10^9^) 1 billion CFU/day	4 weeks
Pineton De Chambrun 2015 [84]	179 *	*S. cerevisiae*	(4 × 10^9^) capsule 4 billion CFU/day	8 weeks
Gade and Thorn 1989 [85]	58	*Streptococcus faecium*	(6.4 × 10^7^) tablet 64 million CFU/day	4 weeks
Yoon 2014 [86]	49	6-strain	(1 × 10^10^) capsule 10 billion CFU/day	4 weeks
Ko 2013 [87]	26	7-strain	(1 × 10^10^) capsule 10 billion CFU/day	8 weeks
Ki Cha 2012 [88]	50	7-strain	1 × 10^10^/day (5 billion per capsule) 10 billion CFU/day	8 weeks
Jafari 2014 [89]	108 *	4-strain	(4 × 10^9^) per capsule—taken twice daily = 8 billion CFU/day	4 weeks
Enck 2008 [90]	297 *	2-strain liquid *Escherichia coli* (DSM 17252) and *Enterococcus faecalis* (DSM 16440)	(1.5–4.5 × 10^7^) liquid 10–30 drops (20 drops = 3–9 × 10^7^) 30–90 million CFU/day	8 weeks
Lorenzo-Zúñiga 2014 [78]	84 total (27 low dose)	3-strain = two *Lactobacillus plantarum* (CECT7484 and CECT7485) and one *Pediococcus acidilactici* (CECT7483)	(3.6 × 10^9^) capsule = 3.6 billion CFU/day	6 weeks
Lorenzo-Zúñiga 2014 [78]	84 (28 high dose)	3-strain	(1.3 × 10^10^) capsule = 13 billion CFU/day	6 weeks
Hong 2009 [91]	70	4-strain	(4 × 10^10^) powder = 40 billion CFU/day	8 weeks
Williams 2009 [79]	56	4-strain	(2.5 × 10^10^) capsule 25 billion CFU/day	8 weeks
Sisson 2014 [92]	186 *	4-strains	(1 × 10^10^ at 1 mL/kg) 50 mL contains 10 billion CFU 1 mL = 200 million CFU (NB: average adult female = 70 kg, average male = 80 kg; therefore = 14–16 billion CFU/day)	12 weeks
Niedzielin 2001 [93]	40	*L. plantarum* 299ve	(2 × 10^10^) Liquid 20 billion CFU/day	4 weeks

(*) Identifies largest trials by sample size. CFU: colony forming unit.

**Table 2:** Summary of evidence on dietary modifications in inflammatory bowel syndrome (IBS), adapted from Tuck and Vanner, 2016 [99]. FODMAP: fermentable oligo-, di-, and monosaccharides, and polyols; SIBO: small intestinal bacterial growth.

Diet	Dietary Modifications	Effectiveness
The National Institute for Health and Care Excellence (NICE) eating pattern recommendation	Regular meal pattern, adequate fluids, limited caffeine, alcohol, fat, fizzy drinks, reduced insoluble fibre but increased soluble fibre	Inconclusive
Modified fibre	Increase/decreased intake of dietary fibre (diet or supplements)	Positive effects with psyllium, but not bran
Low-FODMAP	Short-term (maximum 8 weeks) reduced intake of foods high in fermentable carbohydrates (fruits, vegetables, grains, and dairy) followed up by re-challenge	Mostly positive, risk if used long-term
SIBO	Diet low in fermentable foods. Variable guidelines, largely non-evidence based	Lacks scientific evidence
Specific carbohydrate	Exclusion of refined sugars and complex carbohydrates (all grains, potatoes, milk, processed meat); variable guidelines	Only preliminary data
Paleo	Restricted intake of all grains, legumes, potatoes, dairy, high fibre intake with lean non-domesticated meats and non-cereal plant-based food	Lacks scientific evidence
Gluten-free	Excludes all gluten-containing grains (wheat, barley, rye, oats)	Often positive, but inconclusive
